# The impact of obesity on surgical outcomes in patients undergoing emergency laparotomy for high-risk abdominal emergencies

**DOI:** 10.1186/s12893-022-01466-6

**Published:** 2022-01-15

**Authors:** Woubet Tefera Kassahun, Matthias Mehdorn, Jonas Babel

**Affiliations:** Department of Visceral, Transplantation, Thoracic and Vascular Surgery, University Hospital of Leipzig, Liebig Strasse 20, 04103 Leipzig, Germany

**Keywords:** Obesity, High-risk emergency, Surgery, Morbidity, Mortality

## Abstract

**Background:**

Obesity has been shown to increase the rates of morbidity and occasionally mortality in patients undergoing nonbariatric elective surgery. However, little is known about the impact of obesity on outcomes after surgery for high-risk abdominal emergencies.

**Methods:**

A single-center retrospective evaluation of outcomes in high-risk abdominal emergency patients categorized by body mass index (BMI) was conducted. Patient demographics, comorbidities, and operative details were analyzed. Patients with normal weight (BMI 18.5–24.9) served as comparators. Multivariable linear and logistic regression analyses were performed to assess the impact of obesity on surgical outcomes.

**Results:**

In total, 886 patients with BMI < 18.5 (underweight; n = 50), 18.5–24.9 (normal weight; n = 306), 25–29.9 (overweight; n = 336) and ≥ 30 (obese; n = 194) based on the World Health Organization (WHO) weight classification criteria met the inclusion criteria. Compared to normal-weight patients, patients with overweight and obesity were older and more likely to be male. The rates of comorbidity (100% vs 91.2%, p =  < 0.0001), morbidity (77.8% vs 65.6%, p = 0.003), and in-hospital mortality (44.8% vs 30.4%, p = 0.001) were all higher in patients with obesity than in normal-weight patients. Patients with obesity had an increased intensive care unit length of stay (ICU LOS) (13 days vs 9 days, p = 0.019) and hospital LOS (21.4 days vs 18.1 days, p = 0.081) and prolonged ventilation (39.1% vs 19.6%, p = 0.003). As BMI deviated from the normal range, the morbidity and mortality rates increased incrementally, with the highest morbidity (87.9%) and mortality (54.5%) rates observed in morbidly obese patients (BMI ≥ 40).

**Conclusions:**

Patients with obesity were the most likely to have coexisting conditions, experience postoperative complications, and die during the first admission following EL for high-risk abdominal emergencies.

## Introduction

Worldwide, the prevalence of obesity is increasing [1]. Obesity is often associated with various comorbid conditions, including type II diabetes mellitus, hypertension, stroke, and coronary artery disease, and thus, an increased risk of mortality [2, 3]. For every 5-unit increase in body mass index (BMI) above 25 kg/m^2^, overall mortality increases by 30% [4]. Excess mortality in patients with BMI above the optimum range of 22.5–25 kg/m^2^ is mainly due to vascular comorbidities. Because obesity is increasing in prevalence and is known to increase morbidity and mortality in the general population, it is perceived as a potential risk factor for adverse postsurgical outcomes. However, reports across a wide variety of surgical studies are conflicting; therefore, the effect of obesity on surgical outcomes is not entirely clear. In some studies, obesity was associated with adverse outcomes [5–7]; yet in others, aside from perhaps an increased risk of minor complications, obesity was either not associated with major adverse outcomes or had a protective effect on survival, which is known as the obesity paradox [8–10].

Emergency laparotomy/laparoscopy (EL) is occasionally performed in patients who are obese, and the surgical outcome can potentially be impacted by the weight burden. In addition, performing any surgical procedure in obese patients in emergency situations is technically challenging, and thus, the risk for the development of various complications is high [6, 11]. For elective surgery, extensive interdisciplinary preoperative management, such as optimization of cardiovascular and pulmonary function and weight reduction, can be initiated, depending on the perceived risk of postoperative complications. Such practices cannot be fully implemented when patients require EL. Thus, optimum preoperative management for obese patients in high-risk emergency situations is almost impossible.

Unlike those in elective surgery populations, there are few, if any, studies highlighting the impact of obesity on surgical outcomes in this unique patient population.

Outcomes of EL in these patients need to be further evaluated. We therefore initiated the present study. Our hypothesis was that despite the existence of the possible obesity paradox, patients with obesity would have increased morbidity and mortality rates following surgery for high-risk abdominal emergencies. To test this hypothesis, we conducted a retrospective study of prospectively collected data examining the outcomes of 886 patients who required EL due to high-risk abdominal emergencies.

This was our experience and contribute to improve knowledge about the impact of weight burden on surgical outcomes in this unique patient population, which is useful information that can be used in further research.

## Materials and methods

This study is a retrospective review of our institutional database (2012–2019). An evaluation of postoperative morbidity and in-hospital mortality in high-risk abdominal surgical emergency patients categorized by BMI was conducted. Weight classification was assigned based upon BMI (kg/m^2^) and as defined by the World Health Organization (WHO) [12]; with BMI cutoff points for underweight (< 18.5), normal weight (18.5–24.99), overweight (25–29.99), and obese (≥ 30).

All underweight, overweight, and obese patients undergoing surgery due to high-risk abdominal emergencies from 2012 to 2019 at our institution were included in the analysis, with normal-weight patients (BMI = 18.5–24.99) as the reference group.

Patients who underwent organ transplantation, EL due to complications of elective surgery, and EL due to minor surgical emergencies were excluded from the analysis. We excluded patients who underwent organ transplantation and EL due to complications of elective surgery because these patients make up a patient population with a very different risk profile for complications and mortality compared to high-risk primary emergency patients. The inclusion of large numbers of patients with minor emergencies would limit the study because the impact of BMI on outcomes is least likely to be demonstrated in patients undergoing low-risk surgery [8, 13].

EL was defined as abdominal exploration that had to be performed as soon as possible after admission in adults of any age due to an unscheduled abdominal emergency.

High-risk abdominal emergencies were defined as those generally associated with a high risk of in-hospital death due to septic and/or hemorrhagic complications and required EL. Given our primary focus in evaluating mortality, we limited the study population to patients who underwent procedures that had mortality rates of more than or equal to 10%.

For the purpose of this study, we categorized all operation indications into 5 categories and procedures into 11 types.

Demographic variables, preexisting comorbid conditions, type of surgical procedure, and postoperative outcomes were analyzed.

The severity of medical conditions at the time of surgery was evaluated using the American Society of Anesthesiologists physical status classification (ASA, [14]). The Clavien–Dindo classification of surgical complications (CDC, [15]) was used to classify surgical complications. Based on the CDC, the comprehensive complication index (CCI, [16]) was calculated for each patient to evaluate the true overall morbidity burden of a procedure.

The main outcome measures were any morbidity and mortality during the same admission.

### Statistical analysis

Descriptive analyses of baseline patient characteristics and outcomes were performed. Univariate analyses were performed using the chi-square test for categorical variables and the t-test (2-tailed) for parametric continuous variables. Continuous variables are presented as means ± standard deviations (SDs) or medians with ranges (Rs). Categorical variables are shown as percentages of the sample in a given category. The relationships of each independent variable with morbidity and mortality were tested using the chi-square test or t-test where appropriate. Variables that reached statistical significance in the univariate model were then entered into a multivariable logistic regression model to identify independent predictors of morbidity and mortality. Odds ratios (ORs) and their 95% confidence intervals (CIs) are presented for each estimate. A 2-tailed p value ≤ 0.05 was considered statistically significant for all tests.

All statistical analyses were performed using statistical software (SPSS 25.0, SPSS Inc., USA).

This study was approved by the institutional ethics committee review board of the medical faculty of the University of Leipzig in Leipzig, Germany. As this was a retrospective study, the need for informed consent was waived by the ethical committee of the medical faculty of the University of Leipzig. We confirm that all methods are carried out in accordance with the relevant guidelines and regulations of the ethics committee.

## Results

Overall, there were 2791 emergency abdominal surgery cases between 2012 and 2019. Among these, 1905 cases (68.2%) were excluded from the present analysis based on the criteria outlined in the methods section. All 886 patients included in the analysis underwent EL for high-risk abdominal emergencies as defined in the previous section. Of these, 50 (5.6%), 306 (34.5), 336 (37.9%), and 194 patients (21.9%) were categorized as underweight, normal weight, overweight and obese, respectively. Detailed statistical analysis was performed for all patients with overweight and obesity. For statistical analysis, we used normal-weight patients (n = 306) as the reference group. Table 1 provides a summary of the demographic and clinical variables analyzed.

**Table 1:** Patient demographics, and preoperative characteristics

Variable	Normal-weight	Obese	P
	(N = 306)	(N = 194)	
Male	172 (56.4)	103 (52.8)	0.571
Female	133 (43.6)	92 (47.2)	0.311
Age, years, mean ± SD	62.93 ± 19.83	66.31 ± 13.13	0.036
BMI, mean ± SD	22.16 ± 1.65	35.59 ± 7.33	< 0.0001
Coexisting conditions (COCs)	279 (91.2)	194 (100)	< 0.0001
COCs per patient, mean ± SD	4.61 ± 2.85	6.20 ± 2.99	< 0.0001
ASA* ≥ 3	215 (74.1)	168 (88.9)	< 0.0001
Hypertension	160 (52.5)	162 (83.1)	< 0.0001
Atrial fibrillation	76 (24.9)	69 (35.4)	0.012
Diabetes mellitus	51 (16.7)	85 (43.6)	< 0.0001
PAD	65 (21.3)	56 (28.7)	0.059
Chronic kidney disease	45 (15.2)	44 (22.9)	0.031
CNS	59 (19.3)	53 (27.2)	0.040
Coronary artery disease	48 (15.7)	37 (19.0)	0.347
Chronic heart failure	57 (18.7)	49 (25.1)	0.086
Liver cirrhosis	25 (8.2)	18 (9.2)	0.687
OAC	95 (31.1)	86 (44.1)	0.003

*BMI* body mass index, *SD* standard deviation, *COCs* coexisting conditions, *CNS* indicates central nervous system disease and holds for patients with medically documented cerebral vascular accident, transient ischemic attack, or neurological deficit of central origin, *PAD* peripheral artery disease, *OAC* medication with oral anticoagulants and antiplatelet agents, *ASA* the American society of anesthesiologists physical status classification. Numbers in bracket indicate values presented in n (%) by group unless noted otherwise. *Percents may not total 100 due to missing data; P values represent the difference between the obese and normal weight group

Overall, more male patients (56%) than female patients underwent EL; however, the sex distribution between patients who were overweight and obese and those with normal weight was not significantly different. On average, the patients with obesity were 3.5 years older than the normal-weight patients.

Among the coexisting conditions, patients with obesity were more likely to have hypertension (83.1% vs 52.5%, p ≤ 0001), atrial fibrillation (35.4% vs 24.9%, p = 0.012), diabetes mellitus (43.6% vs 16.7%, p ≤ 0.0001) and chronic kidney disease (22.9% vs 15.2%, p = 0.031) than normal-weight patients.

Table 2 provides a summary of the primary indications for EL and the types of surgical procedures. As depicted in this table, the distributions of the primary indications for surgery and the types of surgical procedures were comparable between groups. More than 80% of the surgical procedures were open procedures.

**Table 2:** Primary indications for surgery and index surgical procedures

	Normal-weight	Obese
	(N = 306)	(N = 194)
*Primary indications*		
Perforated viscus	136 (44.5)	84 (43.6)
Mesenteric ischemia	53 (17.4)	43 (22.1)
Bowel obstruction	79 (26.7)	40 (20.5)
Hemorrhage	28 (9.1)	12 (6.3)
Miscellaneous	10 (3.3)	15 (7.7)
*Index procedures*		
Closure of viscus organ	61 (20.6)	22 (11.2)
Right colectomy including subtotal resection	34 (11.2)	31 (16.1)
Multivisceral procedures	40 (13.5)	21 (10.9)
Small bowel resection	43 (14.5)	20 (10.4)
Laparotomy with extensive adhesiolysis	45 (15.2)	19 (9.9)
Hartmann`s procedure	25 (8.4)	22 (11.5)
Surgery for complicated cholecystitis	12 (4.1)	19 (9.9)
Hemostasis	15 (5.1)	7 (3.6)
Laparotomy only	8 (2.7)	10 (5.2)
Colectomy unspecified	6 (2.0)	6 (3.1)
Miscellaneous	16 (5.3)	18 (9.3)
*Initial access to abdomen*		
Laparoscopy	77 (25.2)	60 (30.9)
Laparotomy	229 (75.0)	134 (69.1)
Conversion	34 (44.2)	27 (45.0)

Numbers in bracket indicate values presented in n (%) unless noted otherwise

The surgical outcomes are summarized in Table 3. Operative times were significantly longer among patients with obesity than among patients with normal weight (≥ 120 min; 55% vs 37.5%, p = 0.0001). The overall postoperative complication rate was significantly higher in patients with obesity than in normal-weight patients (77.8% vs 65.6%, p = 0.003). This was also reflected in the mean number of complications per patient (5.70 ± 3.61 vs 4.70 ± 3.27, p = 0.009) and the CCI (64.38 ± 36.29 vs 50.58 ± 37.02, p < 0.0001).

**Table 3:** Comparison of outcomes

Variable	Normal-weight	Obese	P
	(N = 306)	(N = 194)	
Operative time > 120 min	111 (37.5)	107 (55.4)	0.0001
Complications overall	200 (65.6)	151 (77.8)	0.003
Complications per patient, mean ± SD	4.70 ± 3.27	5.70 ± 3.61	0.009
CCI, mean ± SD	50.67 ± 37.04	64.4 ± 36.3	< 0.0001
Bleeding events	58 (19.0)	60 (30.8)	0.003
Major bleeding	30 (10.0)	45 (23.3)	< 0.0001
BPT	62 (20.3)	63 (32.3)	0.003
Surgical site infection	95 (31.1)	79 (40.5)	0.032
Anastomotic leaks	35 (11.5)	24 (12.3)	0.735
Pneumonia	71 (23.3)	57 (29.2)	0.137
Respiratory impairment	103 (33.7)	106 (54.6)	< 0.0001
Thromboembolic events	60 (19.9)	35 (18.1)	0.633
Liver failure	53 (17.4)	64 (32.8)	< 0.0001
Acute renal failure	82 (27.0)	86 (44.3)	< 0.0001
URLs	97 (32.6)	80 (41.0)	0.050
ICU	221 (72.5)	160 (82.1)	0.014
ICU-LOS, days, mean ± SD	9 ± 15	13 ± 19	0.019
ICU-LOS, days, median (R)	4 (1–127)	6 (1–135)	
MV ≥ 24 h	58 (19.6)	75 (39.1)	0.003
In-hospital mortality	93 (30.4)	87 (44.8)	0.001
LOS, days, mean ± SD	18.05 ± 18.9	21.43 ± 24.2	0.081
LOS, days, median (R)	12 (1–154)	14 (1–200)	

Surgical site infection is defined as being contained within the skin or subcutaneous tissue (superficial), or involving the muscle and /or fascia (deep); acute renal failure was considered if it required dialysis; *CCI* the comprehensive complication index, *BPT* blood product transfusion, *URL* unplanned relaparotomy, *ICU* intensive care unit requirement, *MV* mechanical ventilation defined as ventilation at any time during hospitalization and applies for all patients who required ventilation beyond the operation room, *DMV* duration of mechanical ventilation, *LOS* length of hospital stay defined as the time from the date of the initial admission to the date of discharge, transfer to external services, or death, which ever came first

Although the rates of anastomotic leaks and pneumonia were higher in patients with obesity than in normal-weight patients, the difference was not statistically significant.

Bleeding, surgical site infection, acute renal failure, and liver failure occurred more frequently in patients with obesity than in normal-weight patients. This difference was statistically significant. Overall, the percentage of every analyzed comorbid condition and complication tended to increase with increasing BMI with 1 exception; thromboembolic events that included deep venous thrombosis, pulmonary embolism, myocardial infarction, ischemic stroke and systemic embolism declined slightly but not significantly in patients with obesity.

Unplanned relaparotomy (URL) was performed more frequently in patients with obesity than in normal-weight patients (41% vs 32.6%, p = 0.05), and the mean number of URLs per patient was also higher in patients with obesity than in patients with normal weight (1.93 ± 1.27 vs 1.53 ± 0.89, p = 0.015).

Relatively high percentages of patients were admitted to the intensive care unit (ICU) in both the obese and normal-weight groups. However, the proportion of patients who required ICU admission was significantly higher in the obese group than in the normal-weight group (82.1 vs 72.5, p = 0.014). In addition, patients with obesity were more likely to be ventilator dependent for more than 24 h than normal-weight patients (39.1% vs 19.6%, p = 0.003).

Eighty-seven (44.8%) of the 194 patients in the obese group and 93 (30.4%) of the 306 patients in the normal-weight group died after surgery without being discharged from the hospital. This difference was statistically significant (p = 0.001). The hospital length of stay (LOS) was longer in the obese group than in the normal-weight group (median 14 vs 12, p = 0.081).

**Fig. 1:**
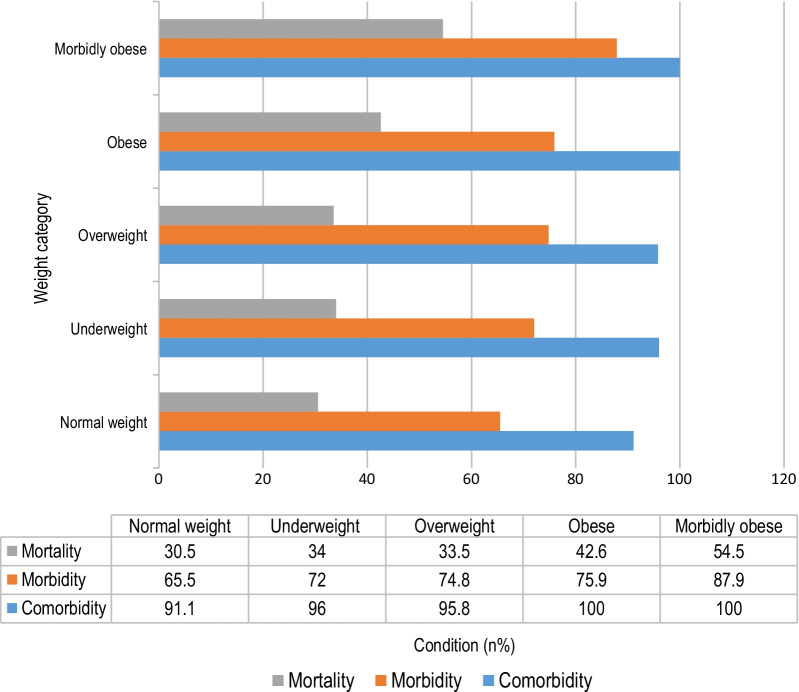
Coexisting conditions and trends of main outcomes by weight category. Normal weight (n = 306), BMI 18.5–24.9; underweight (n = 50), BMI < 18.5; overweight (n = 336), BMI 25–29.9; obese (n = 161), BMI, 30–39.9; morbidly obese (n = 33), BMI ≥ 40

Owing to the significant variance in major adverse events according to BMI, we stratified our entire cohort by BMI and analyzed the main results. The results are shown in Fig. 1.

For patients undergoing EL for high-risk abdominal emergencies, comorbidities, morbidity and mortality rates differed by BMI category.

Overall, patients with obesity were the most likely to have coexisting conditions (100%), to experience postoperative complications (77.8%) and to die during the first admission (44.8%). As BMI deviated from the normal range, the morbidity and mortality rates increased incrementally, with the highest morbidity (87.9%) and mortality (54.5%) rates observed in morbidly obese patients (BMI ≥ 40)).

The results of the uni- and multivariate regression models are presented in Tables 4 and 5.

**Table 4:** Risk of postoperative complications associated with a specific comorbidity

Risk factor	Unadjusted		Adjusted	
	OR (95% CI)	p value	OR (95% CI)	p value
Age	2.18 (1.45–3.28)	< 0.0001	1.87 (1.07–3.30)	0.028
ASA	5.61 (3.46–9.09)	< 0.0001	2.55 (1.41–4.62)	0.002
BMI	2.81 (1.19–3.74)	0.005	1.27 (0.74–2.19)	0.389
Hypertension	1.76 (1.18–2.62)	0.005	0.94 (0.51–1.74)	0.851
Atrial fibrillation	2.12 (1.32–3.39)	0.002	1.01 (0.55–1.86)	0.967
PAD	4.33 (2.22–7.33)	< 0.0001	2.02 (1.09–4.46)	0.028
Chronic renal failure	2.01 (1.14–3.55)	0.015	0.97 (0.45–2.07)	0.935
Chronic heart failure	2.07 (1.21–3.52)	0.007	1.02 (0.51–2.08)	0.940
Liver cirrhosis	9.20 (2.19–38.64)	0.0002	5.23 (1.13–24.27)	0.035
URL	15.86 (7.53–33.39)	< 0.0001	13.06 (6.06–28.15)	< 0.0001

*OR* odds ratio, *CI* confidence interval, *ASA* American Society of Anesthesiologists Physical Status classification, *BMI* body-mass index, *PAD* peripheral artery disease, *URL* unplanned relaparotomy

**Table 5:** Risk of in-hospital mortality associated with a specific comorbidity and postoperative complication

Risk factor	Unadjusted		Adjusted	
	OR (95% CI)	p value	OR (95% CI)	p value
Age	1.74 (1.19–2.52)	0.004	1.74 (0.64–4.78)	0.280
ASA	23.4 (7.28–75.25)	< 0.0001	1.45 (0.16–12.90)	0.741
BMI	1.87 (1.28–2.74)	0.001	0.97 (0.39–2.44)	0.950
Hypertension	1.92 (1.28–2.89)	0.002	0.79 (0.26–2.41)	0.678
Atrial fibrillation	2.22 (1.49–3.32)	< 0.0001	1.42 (0.50–4.01)	0.512
Peripheral arterial disease	3.82 (2.48–5.90)	< 0.0001	1.57 (0.57–4.35)	0.387
Chronic renal failure	2.54 (1.59–4.05)	< 0.0001	1.34 (0.47–3.86)	0.583
Chronic heart failure	1.81 (1.16–2.8)	0.008	0.89 (0.33–2.42)	0.821
Liver cirrhosis	10.6 (4.61–24.62)	< 0.0001	3.03 (0.27–33.85)	0.396
Bleeding events	9.04 (5.60–14.60)	< 0.0001	1.46 (0.34–6.27)	0.613
Major bleeding	15.63 (7.95–30.75)	< 0.0001	0.97 (0.19–4.96)	0.966
BPT	11.73 (7.19–19.13)	< 0.0001	0.63 (0.15–2.66)	0.523
Anastomotic leaks	3.77 (2.13–6.68)	< 0.0001	5.49 (1.57–19.14)	0.008
Pneumonia	8.02 (5.09–12.63)	< 0.0001	2.90 (1.13–7.49)	0.028
Thromboembolic events	8.11 (4.81–13.69)	< 0.0001	1.74 (0.62–4.86)	0.289
Liver failure	296.40 (71.28–1232.45)	< 0.0001	34.46 (6.45–184.06)	< 0.0001
Acute renal failure	83.67 (45.03–155.47)	< 0.0001	8.28 (2.67–25.64)	< 0.0001
Reoperation	4.38 (2.94–6.52)	< 0.0001	1.32 (0.50–3.45)	0.578
Ventilator dependence	3.30 (1.93–5.62)	< 0. 0001	0.45 (0.15–1.36)	0.157

*OR* odds ratio, *CI* confidence interval, *ICU* intensive care unit stay, *BPT* blood product transfusion; ventilator dependence was defined as ventilation longer than 24 h at any time during hospitalization

In the univariate analyses, 10 and 19 demographic and clinical variables were significantly predictive of morbidity and all-cause in-hospital mortality, respectively. Of these, only 5 variables (age, ASA, peripheral artery disease [PAD], liver cirrhosis, and URL) and 4 variables (anastomotic leakage, pneumonia, acute renal failure, and liver failure) remained independent predictors of morbidity and mortality, respectively, in the multivariate models. We note that obesity was associated with increased morbidity and mortality risks in the unadjusted model but not in the multivariate logistic regression model.

## Discussion

We conducted a retrospective single-center study to analyze the association between obesity and postoperative adverse outcomes following EL for high-risk abdominal emergencies. We found that patients with obesity had significantly higher rates of complications and mortality than nonobese patients. Comorbidities, morbidity and mortality were lowest in the normal-weight group.

Our findings suggest that BMI is an important clinical factor that influences outcomes in high-risk abdominal emergency patients undergoing surgery that cannot be personalized and optimized to their weight in emergency situations.

Almost all postoperative-specific complications were found to have increased rates among patients with obesity. Other studies also support that obesity is associated with an increased complication rate in surgical patients. Giles et al. [17] studied patients undergoing aortic aneurism repair and showed that the rate of complications was 2 times higher in patients with obesity than in normal-weight patients, and patients with obesity had significantly higher mortality. Liu et al. [18] performed a systematic review of studies in trauma patients and showed that obesity was associated with increased risks of postoperative complications and mortality. For general surgery procedures, Yanquez et al. [19] found an increased risk of complications in patients with obesity.

The comorbidity rate was significantly higher in patients with obesity than in normal-weight patients. In a separate analysis, many of the comorbid conditions predicted the risk of specific postoperative complications. In decreasing order of value, these factors included URL, liver cirrhosis, PAD, ASA class ≥ 3, age ≥ 70 years, atrial fibrillation, chronic heart failure, chronic renal failure, BMI, and hypertension. In the multivariable logistic regression analysis, age, ASA, PAD, liver cirrhosis and URL remained significant predictors of complications. It is widely accepted that BMI above the normal range affects many comorbid conditions, particularly cardiovascular and renal diseases [3, 4]. Thus, by extension, the increased complication rate observed in our study seems to be a sequela of comorbid conditions associated with obesity. Furthermore, patients with obesity undergoing surgery for high-risk abdominal emergencies required prolonged mechanical ventilation, defined as postoperative intubation for longer than 24 h, and thus a prolonged bedridden hospital stay. Therefore, these patients are likely to develop complications that subsequently contribute to mortality. This is in agreement with the results of other studies that reported an increased frequency of prolonged ventilation in obese patients undergoing elective surgical procedures [20]. The incidence of pneumonia did not differ between obese and normal-weight patients; however, postoperative respiratory impairment that resulted in ICU admission was frequent in patients with obesity, similar to the findings of other researchers [21].

Specific data on outcomes after surgery for high-risk abdominal emergencies were not available. In a relatively small retrospective study, Ferrada et al. [22] found no significant increase in mortality in patients with obesity undergoing emergency surgery. Although a consensus definition of obesity has been established by the WHO, these authors defined patients as nonobese and obese and included underweight patients in the nonobese and overweight patients in the obese group without accurate categorization of patients according to their BMI. Mortality in patients with obesity following emergency surgery cannot be analyzed in this context, as the occurrence of major adverse events differs based on BMI.

In contrast, the previously reported finding that patients with obesity suffer a higher rate of mortality in aortic repair [17], trauma [18], and elective general surgeries [23] was verified in our analysis of high-risk abdominal emergency patients. This demonstrated that in-hospital mortality in patients undergoing EL for high-risk emergencies varied by weight classification. There was a gradual increase in mortality with increasing weight class, where patients with BMI ≥ 40 fared the worst. This indicates that even small changes in weight can affect outcomes. Our multivariate regression analyses of the factors identified to be significantly different in obese patients found that the ORs for pneumonia, anastomotic leakage, acute renal failure and liver failure were 2.9, 5.5, 8.3 and 34.5, respectively. It is likely that these complications are responsible for the absolute excess mortality in patients with BMI ≥ 30. However, we cannot infer a causal relationship with these data.

The operative time and LOS were longer, and the ICU admission rate was higher in patients with obesity than in normal-weight patients. This indicates that in addition to its clinical significance in morbidity and mortality, obesity may have a widespread impact on overall treatment cost. Therefore, although impossible to implement in emergency situations, optimizing nutrition and weight in the general population would have a decisive role in minimizing complications and cost-related variables, such as LOS, and indirectly improve outcomes (in the long term), even in patients undergoing surgery due to high-risk emergencies.

The underlying mechanisms of these adverse events (i.e., morbidity and mortality) following surgery for high-risk abdominal emergencies are not entirely clear; however, there is evidence that energy use in patients with obesity, especially those in the higher BMI category (BMI ≥ 35; 40% of our patients with obesity), is inefficient, and underlying metabolic excess leads to hyperbolic inflammatory responses, oxidative stress, and further metabolic dysfunction and immunosuppression. As a result, these patients are not fit to handle the extreme stress imposed by surgery in emergent situations and thus experience more adverse events [9, 24, 25].

Overall, our results suggest that BMI itself was not an independent factor predictive of in-hospital mortality following EL for high-risk abdominal emergencies. However, the percentage of every analyzed comorbid condition and complication tended to increase with increasing BMI and there was a gradual increase in mortality with increasing weight class, where patients with BMI ≥ 40 fared the worst. Therefore, obesity may predispose patients to mortality through its impact on numerous coexisting conditions and postoperative complications following EL for high-risk abdominal emergencies. The lack of a significant statistical association between increasing BMI and in-hospital mortality in the multivariate model probably reflects the relatively small sample size, especially in the highest BMI categories, rather than biologic reality.

Compared to recent data in the literature [26, 27] that reported on the association of obesity with morbidity and mortality in trauma patients, the mortality rate in our cohort was notably high. Given the very different risk profiles for complications and mortality of trauma patients, however, the high mortality rate indicated in this study is relative and attributable to the risk-based approach of patient selection. In trauma patients, the injury mechanism to the abdomen is either blunt or penetrating. We included only consecutive multimorbid patients with high-risk emergencies, such as mesenteric ischemia and viscous organ perforation, mostly related to chronic illnesses, where the acute insult is greater. These patients are at high risk of procedural adverse events and tend to have septic complications with multiple organ dysfunction that inevitably lead to death [28, 29]. If we consider our entire primary emergency cohort including those patients with minor emergencies, the overall mortality rate is less than 15%, which is within the range of mortality rates reported in the literature [30–32].

Our study has limitations that are inherent to a retrospective study. First, this was a retrospective study and was therefore subject to reviewer error and data miscoding, and certain details important to the analysis of outcomes were missing. We did not include lifestyle factors such as smoking, alcohol consumption, lack of physical activity, dietary factors and medication compliance in the analysis, which could have provided alternative explanations for our findings; unfortunately, data on these variables were not consistently recorded in our database. Second, our study was a single-center study; our findings may therefore not be generalizable to other centers and institutions at large. Finally, we categorized BMI into underweight, overweight and obese using normal-weight patients as the reference group, but a detailed statistical analysis for underweight patients was not performed. In addition to focusing on obese patients, delineating underweight patients is necessary in the identification of major outcomes because underweight is associated with additional individual health risks. However, the approach we undertook was necessary owing to the small sample size in the underweight group (only 50 patients), which would have limited the statistical power.

Despite these limitations, our study provides an important evaluation of the impact and outcomes associated with obesity in a population of patients undergoing surgery due to high-risk abdominal emergencies.

## Conclusions

We retrospectively analyzed a cohort of patients who underwent EL for high-risk abdominal emergencies based on BMI. We found that patients with obesity were the most likely to have coexisting conditions, experience postoperative complications, and die during the first admission. The rates of pneumonia, anastomotic leakage, acute renal failure, and liver failure were all higher in obese patients and were found to be associated with an increased risk of in-hospital mortality, making this an important relationship to consider. Thus, although BMI itself was not an independent factor predictive of in-hospital mortality, patients with obesity were the most likely to have coexisting conditions, experience postoperative complications, and die during the first admission following EL for high-risk abdominal emergencies. The lack of a significant statistical association between increasing BMI and mortality in the multivariate model in our study probably reflects the relatively small sample size, especially in the highest BMI categories, rather than biologic reality.

## Data Availability

The datasets generated and/or analyzed during the current study are not publicly available due to internal institutional restrictions but are available from the corresponding author on reasonable request and with the permission of the institution where the data was generated.

## Abbreviations

EL: Emergency laparotomy
BMI: Body mass index
COCs: Coexisting conditions
ASA: The American Society of Anesthesiologists Physical Status classification
ICU: Intensive care unit
MV: Mechanical ventilation
PAD: Peripheral arterial disease
BPT: Blood product transfusion
URL: Unplanned relaparotomy
CDC: Clavien–Dindo classification of complications
CCI: Comprehensive complication index
LOS: Length of hospital stay
